# Some Points in Urine Examination

**Published:** 1920-03-06

**Authors:** 


### SMALLER MEDICAL FALLACIES.

### Some Points in Urine Examination.

The main reason which brings these few notes into existence is the fact that, although in the vital early stages of medical training all are taught more or less assiduously the classical tests which can and should be performed in the small clinical laboratory attached to the ward and consulting-room, yet so many of us come to take these small experiments and the results obtained, as being unfailingly trustworthy, liable to relatively few fallacies, and withal sufficient for ordinary clinical needs. Not only do we later come to use a stereotyped few, but in doing so are apt to forget that there is an enormous amount of valuable information to be gained from the things which happen in our test tubes or under our microscopes—quite apart from the actual positive or negative results, which the classical tests may give. There are many extremely simple methods which are relatively rarely employed at home, and the valuable time often wasted in awaiting the report on a specimen sent to some central laboratory is a matter for considerable regret.

### Urinary Deposits.

Let us first consider the simple urinary deposits. If a centrifuge be used so much the better, but a satisfactory sediment can be readily obtained in a tall specimen glass on standing. If the supernatant urine be poured into another receptacle a single drop of this deposit can be transferred by a pipette to a clear side. Then with a glass rod one drop of a normal saline solution of methylene blue or neutral red may be placed, not in it, but near it, on the side. The two drops gently mixed may be lightly covered by a cover-slip and the surplus fluid removed by gentle blotting. Examined with a $\frac{1}{6}$-inch objective, the cellular elements and nuclei will be found to have stained particularly, and the paler colour in liquid background makes an excellent contrast with the crystals. Bacteria are lightly stained and still retain their shape, formation, and motility. As the diaphragm of the microscope is gradually closed down a perfect view of the deposited elements may be obtained. This simple fresh staining gives excellent results, but is surprisingly neglected in practice.

Most of the findings to be experienced are too well known to call even for reference, but remember the sequence; excess of oxalate crystals, "oxaluria" (following acid dyspepsia), irritation of the urinary passages by the crystals, leading to the presence of nucleo-proteid or even spermatozoa in the urine and an erroneous diagnosis of albuminuria when the heat test alone is applied. "Coffin lids" of ammonio-magnesin phosphate are common in ammoniacal urine and of no import after a urine has decomposed on standing, but if found in *fresh* urine they indicate cystitis or some cause at least of decomposition *within* the urinary tract. Do not forget the rarity but also the importance of tyrosin and leucin crystals, which are readily observed by the seeing eye and indicate atrophic liver changes.

With regard to cell elements, there is not nearly that difficulty and indefiniteness which some are apt to think. True degenerate cells, particularly leucocytes and renal cells, look much alike, but a very little further search will show whether one or both are present, and in practice with fresh specimens some typical cells are sure to be found. We see the large squamous cell, an inch square under the $\frac{1}{12}$ inch; the two types of bladder cell, the superficial ones convex in their free surfaces and with concavities on their underside, into which fit the tailed oval cells of the deeper layer. Cells of this deep layer in any great number indicate ulceration. Renal cells, if fresh, conform very well to the classical types of the book. If there be excoriation of the renal *pelvis* in pyelitis, calculus, or tubercle, a characteristic pelvic cell may be noted and is important. Long, narrow, with a single nucleus and a slender wavy tail, this cell is very characteristic, easily seen, and, in quantity, diagnostic of locality and a pathological condition. The subject of new growths and fragments derived from them we will pass. They are not easy to detect, and the expert is necessary to distinguish between them and clumps of epithelial cells. The simple red blood cell must not be forgotten, however; extraordinarily often they are missed, possibly because in a concentrated urine they crenate or crumple up; in a dilute one they bake and appear as ghosts. Apart from menstruation they are always pathological.

### Casts and Pseudo-Casts.

Casts, their differentiation and their significance, make a subject too well treated in the text-books to call for comment here, but one small point may be mentioned. Mostly mucus in the urine has no significence, but it is extraordinarily easy to mistake mucus for renal casts. Often the mucus has a cylindrical form, and also, when a cover-slip is first pressed down upon them, there is a slipping movement and the mucus is pressed out lengthwise in a series of peculiarly cast-like streaks. In that all these lie in the one axis and are present in great numbers and of variable diameter, the mistake is not made after the fallacy is once experienced, but in view of the serious import of a similar number of tube casts, it is important to be aware of the possible error.

If we run through some of the fallacies of ordinary test-tube experiments there are some in even those most commonly employed. When adding nitric acid to a concentrated urine, remember the white ring may be due to urea nitrate and that a patient's urine after copaiba also gives a beautiful artefact due to a precipitation of the resin. Iodide passed in the urine gives a positive result to the guiac test for blood or pus, also some tap-waters will do this if organic matter be present. Saliva or sputum in the urinary receptacle is also a notorious source of error. Coming next to glycosuria, Fehling's test is simple enough in itself, and detects extremely minute quantities of sugar; but again it must be noted that the presence of albumin interferes with it and the latter must first be coagulated and removed. Among drugs sometimes present in the urine there are chloral, chloroform, glycerine, carbolic acid, salicylates, and urotropine, all liable to produce reduction. Lastly, if the urine be not fresh and is ammoniacal from decomposition, the presence of free ammonia may easily prevent the precipitation of cuprous oxide and mask the whole result.

So might one proceed to raise many more well-known but often forgotten fallacies in simple urine work, and the critic will say we have but one-tenth fulfilled our task of demonstration that such things abound, but these few words are notes and nothing more. Perhaps as such they may have achieved a limited but somewhere useful purpose.

